# Whole-genome sequencing reveals a unique outbreak of methicillin-resistant *Staphylococcus aureus* clone USA400/J in Japan

**DOI:** 10.1016/j.infpip.2025.100506

**Published:** 2025-12-13

**Authors:** Takuma Yoshida, Yuka Yamagishi, Hiroshi Kaneko, Shunsuke Takadama, Hiroshige Mikamo, Hidemasa Nakaminami

**Affiliations:** a Department of Clinical Microbiology, School of Pharmacy, Tokyo University of Pharmacy and Life Sciences, Hachioji, Tokyo, Japan; b Department of Clinical Infectious Diseases, Kochi Medical School, Kochi University, Kochi, Japan; c Department of Clinical Infectious Diseases, Aichi Medical University, Nagakute, Aichi, Japan

**Keywords:** Methicillin-resistant *Staphylococcus aureus*, Neonatal intensive care unit, Outbreak, Whole-genome sequencing, USA400/J

## Abstract

**Objectives:**

Panton-Valentine leukocidin (PVL)-negative, sequence type 1-staphylococcal cassette chromosome *mec* (SCC*mec*) type IV (ST1-IV) methicillin-resistant *Staphylococcus aureus* (MRSA) is prevalent in Japanese hospitals. The PVL-negative ST1-IV MRSA strain is known as USA400/J in Japan. In this study, we analysed an MRSA outbreak at a Japanese university hospital using conventional methods, SCC*mec* typing, pulsed-field gel electrophoresis (PFGE) and whole-genome sequencing (WGS).

**Methods:**

We analysed 12 MRSA strains isolated from the neonatal intensive care unit of Aichi Medical University Hospital. SCC*mec* typing and gene detection were performed using polymerase chain reaction. Molecular epidemiological analyses were performed using multi-locus sequence typing and PFGE. Genome-based phylogenetic analyses were performed for some strains.

**Results:**

The isolated MRSA strains were classified as ST1-IV (*N* = 4), ST8-IV (*N* = 1), ST764-II (*N* = 6) and ST89-V (*N* = 1). PFGE analysis showed that the ST1-IV and ST764-II strains exhibited high homology within their clones. Phylogenetic analysis based on the genomes of the USA400/J strains isolated in this study and ST1-IV isolates from overseas showed that clonal complex 1-SCC*mec* type IV (CC1-IV) strains isolated in Japan formed a unique cluster that was distinct from the ST1-IV strains from overseas. This suggests that CC1-IV evolved and spread independently in Japan.

**Conclusion:**

These findings highlight the need for increased surveillance and infection control measures that specifically target USA400/J.

## Introduction

*Staphylococcus aureus* can cause serious opportunistic infections in clinical practice. Although advances have been made in the treatment of these pathogens, the emergence of antimicrobial-resistant bacteria, particularly methicillin-resistant *Staphylococcus aureus* (MRSA), is a major concern worldwide. MRSA is a common pathogen associated with both hospital- and community-acquired infections. Premature, very-low-birthweight and critically ill babies are particularly vulnerable to infection because their immune systems are immature and often undergo multiple invasive procedures [1]. MRSA infections increase the risk of neonatal death and the economic burden of prolonged hospitalisation [[2], [3], [4]]. MRSA outbreaks in hospitals, especially neonatal intensive care units (NICUs), are a global problem [5]. Factors contributing to MRSA colonisation in the NICU include medical equipment, the indoor environment and breast milk contamination [[6], [7], [8]]. In addition, the introduction of MRSA by healthcare workers and mothers who are carriers is also considered a potential factor [9]. This is particularly true if healthcare workers are carriers because there is an increased risk of transmission to newborns through treatment and routine contact [8]. MRSA can survive on moist or cold environmental surfaces for long periods of time [10,11]. Therefore, many hospitals closely monitor newborns for MRSA carriage and follow strict sterilisation protocols [8].

In most regions of Japan, the predominant MRSA strains isolated in hospitals carry staphylococcal cassette chromosome *mec* (SCC*mec*) type IV [12,13]. Notably, clonal complex 1-SCC*mec* type IV (CC1-IV) strains, predominantly classified as sequence type 1 (ST1), have recently become dominant. The prototypical sequence type 1-SCCmec type IV (ST1-IV) strain, USA400, is characterised by the production of Panton-Valentine leukocidin (PVL) [14]. However, the majority of ST1-IV strains isolated in Japan are PVL-negative and are considered to be phylogenetically distinct from the classical USA400 lineage [13,15,16]. In response, Japanese guidelines have reclassified PVL-negative ST1-IV strains as ‘USA400/J’, clearly distinguishing them from conventional USA400 strains. Although it lacks PVL, there have been several reports of an increasing number of clinical cases and outbreaks caused by USA400/J. This suggests that many aspects of its pathogenicity and modes of transmission are not well understood [13,[17], [18], [19]]. In recent years, USA400/J outbreaks have frequently occurred in both acute care hospitals and long-term care facilities, posing a significant challenge to infection control in healthcare settings [17,18].

MRSA outbreaks are usually analysed using conventional molecular epidemiology methods, such as SCC*mec* typing and pulsed-field gel electrophoresis (PFGE). However, whole-genome sequencing (WGS) has recently been shown to have high discriminatory power [20]. WGS has been shown to be useful in outbreak analysis because it can characterise pathogens in detail [21]. In addition, WGS has been used in various fields of clinical microbiology, including evolutionary studies and phylogeographic distribution [22,23]. In this study, an MRSA outbreak occurring in the NICU of Aichi Medical University Hospital was analysed using conventional methods and WGS.

## Materials and methods

### Bacterial strains, growth conditions and MRSA identification

The study protocol was approved by the Ethics Committee of Aichi Medical University (2019-028). Informed consent was not required from patients because the study did not involve any clinical interactions. The NICU at Aichi Medical University Hospital had nine beds and treated a total of 2251 patients per year. This equates to an average of 6.2 patients per day, a bed occupancy rate of 68.5%, an average length of stay of 20 days and 102 new admissions. During the outbreak period, the NICU was staffed by physicians, nurses (including midwives), nursing assistants and pharmacists. Each profession provided care in the NICU. A total of 797 samples, including urine and faecal matter, were collected for this study between July 2018 and April 2019. All clinical isolates were identified as *S. aureus* by positive Gram staining, proliferation on mannitol salt agar (BD, New Jersey, USA), coagulase production test (PS LATEX; Eiken Chemical Co., Ltd., Tokyo, Japan) and *nuc* gene detection [24]. The following *S. aureus* strains were used as SCC*mec* type strains: NCTC10442 (type I), N315 (type II), 85/2082 (type III), JCSC4744 (type IV) and WIS (type V) [25]. MRSA isolates were identified using polymerase chain reaction (PCR)-based detection of the *mecA* gene. The MRSA strain N315 was used as a DNA reference standard for PFGE analysis, and the USA400 clone MW2 was used to compare PFGE patterns.

### PCR amplification

PCR assays for the detection of *mecA*, PVL genes (*lukS/F-PV*), arginine catabolic mobile element (ACME) genes (*arcA* and *opp-3*) and the toxic shock syndrome toxin-1 (TSST-1) gene (*tst*) were performed as previously described [26].

### Molecular epidemiological analysis by SCC*mec* typing, multi-locus sequence typing and PFGE

SCC*mec* typing, multi-locus sequence typing (MLST) and PFGE analysis were performed as previously described [[27], [28], [29]]. Briefly, SCC*mec* typing was conducted using the PCR assay, and the isolates were classified into types I–V. The nucleotide sequences of housekeeping genes were determined using ABI 3500 Genetic Analyzer (Thermo Fisher Scientific). Alleles were assigned by comparing the obtained sequences with those deposited in PubMLST online database (https://pubmlst.org/). PFGE DNA patterns were analysed using BioNumerics software version 7.1 (Applied Maths, Sint-Martens-Latem, Belgium), applying the Dice coefficient (1.0% optimisation and 1.0% band tolerance). Strains exhibiting ≥80% similarity in banding patterns were classified as the same pulsotype.

### Genomic analysis

Genomic analysis was performed as previously described with some modifications [30]. Briefly, genomic DNA was extracted using the DNeasy Blood & Tissue Kit (QIAGEN, Hilden, Germany). WGS was performed using DNBSEQ-G400 (MGI Tech Co., Ltd., Shenzhen, China) and NextSeq 2000 (Illumina, Inc., California, USA). A phylogenetic tree based on whole-genome single-nucleotide polymorphisms (SNPs) was constructed using CSI Phylogeny and visualised using FigTree v1.4.4 [31]. This analysis was performed using the default settings. We used 43 previously reported strains of CC1-IV MRSA as reference genomes [15,17,19,[32], [33], [34], [35], [36], [37], [38]]. To ensure high homology, CC1-IV MRSA strains were mapped to MW2 (accession no. BA000033), using Geneious Prime 2019.2.3 (www.geneious.com). SCC*mec* types and MLST profiles of ST1-IV strains (AM15, AM21, AM23 and AM25) were determined using the WGS data. PVL genes were predicted using VirulenceFinder 2.0 (≥90% identity and ≥60% length coverage) [39,40].

## Results

### Patient characteristics and description of outbreak

Twelve MRSA strains were isolated from the NICU of Aichi Medical University Hospital (Table I). These MRSA strains have been detected in infants born at 25–31 weeks of gestation. The PFGE analysis performed at the hospital revealed that the strains exhibited similar patterns. Therefore, an MRSA outbreak was suspected. The 12 strains were isolated over a period of nine months. The first strain was detected in July 2018, and the last strain was detected in April 2019. The continued isolation of each strain from patients throughout this period suggests that the outbreak in the NICU was prolonged. During the outbreak, MRSA was isolated from neonates but not from medical staff. Of the cases, only one presented with symptoms of bacteraemia, which is characterised by fluctuating C-reactive protein (CRP) levels and an absence of fever. The remaining cases were asymptomatic carriers. Following the outbreak, maternity admissions were temporarily restricted to prevent the spread of infection. Patients who required new management for preterm birth or other reasons were transferred to other perinatal care facilities. Meanwhile, as there were no outbreaks of infection in the obstetrics and gynaecology outpatient clinics, wards or maternity units, outpatient care and admission to the obstetrics and gynaecology wards continued as usual. Environmental cultures in the NICU showed that MRSA was isolated from incubators, computers, lights, chairs and other items placed near the affected patients. Therefore, outbreaks due to environmental contamination were suspected, and it was found that the cleaning of high-contact areas and management of shared items were inadequate. The hospital routinely monitored both the volume of alcohol-based hand rub used and compliance with the ‘Five Moments for Hand Hygiene’. The institution consistently met its own monthly targets. Furthermore, no MRSA outbreaks had been reported at the hospital prior to this incident.

**Table I:** Patients’ information

Case	Sex	Gestational age (weeks)	Admission	MRSA	Isolation	Specimen
				Strain no.		
1	Woman	26	8/4/2018	AM14	8/14/2018	Pus
2	Man	25	5/1/2018	AM15	8/14/2018	Nasal discharge
3	Man	31	8/11/2018	AM16	8/21/2018	Nasal discharge
4	Man	25	8/13/2018	AM17	8/21/2018	Nasal discharge
5	Woman	25	8/19/2018	AM18	8/24/2018	Stool
6	Man	25	8/19/2018	AM19	9/4/2018	Stool
7	Woman	37	11/30/2018	AM20	12/2/2018	Stool
8	Woman	37	12/28/2018	AM21	1/29/2019	Stool
9	Man	37	12/27/2018	AM22	1/8/2019	Stool
10	Man	26	1/16/2019	AM23	2/5/2019	Nasal discharge
11	Woman	31	2/2/2019	AM24	2/19/2019	Nasal discharge
12	Man	31	2/19/2019	AM25	2/26/2019	Nasal discharge

MRSA, methicillin-resistant *Staphylococcus aureus*.

### Molecular epidemiological analysis of MRSA isolates

**Figure 1:**
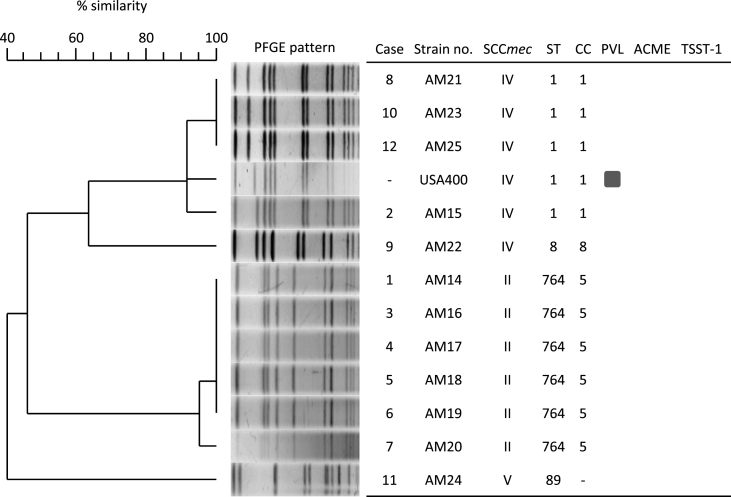
Molecular epidemiological analysis of MRSA isolates from the NICU (*N* = 12) based on PFGE, SCC*mec* type, MLST and virulence genes. The presence of virulence genes is indicated by squares. ST, sequence type; CC, clonal complex; PVL, Panton-Valentine leukocidin; ACME, arginine catabolic mobile element; TSST-1, toxic shock syndrome toxin-1; NICU, neonatal intensive care unit; PFGE, pulsed-field gel electrophoresis; MLST, multi-locus sequence typing; MRSA, methicillin-resistant *Staphylococcus aureus*.

We characterised MRSA isolates using SCC*mec* typing and virulence gene detection (Figure 1). In addition, the genotype of each strain was determined using MLST. We isolated four strains of ST1-IV, one strain of ST8-IV, six strains of ST764-II and one strain of ST89-V. None of the strains carried PVL, ACME or TSST-1. PFGE analysis revealed that ST1-IV and ST764-II strains exhibited high homology within their clones. Therefore, these strains were identified as the cause of the outbreak. ST1-IV strains were phylogenetically closely related to the PVL-positive USA400 but were negative for the characteristic toxin, PVL. Thus, the ST1-IV isolates in this study were USA400/J, as defined by the guidelines for MRSA infection in Japan.

### Genomic analysis

**Figure 2:**
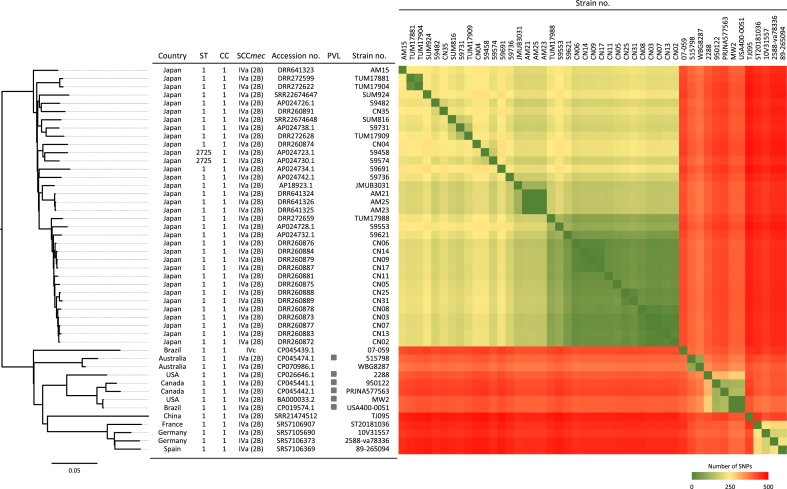
Phylogenetic tree of CC1-IV strains generated using CSI Phylogeny. The scale bar indicates the average number of nucleotide substitutions per site. MW2 was used as the reference genome for the phylogenetic analysis. The presence of PVL genes is indicated by squares. The number of SNP pairs in CC1-IV MRSA strains is shown in the table on the right. ST, sequence type; CC, clonal complex; PVL, Panton-Valentine leukocidin; CC1-IV, clonal complex 1-SCC*mec* type IV; MRSA, methicillin-resistant *Staphylococcus aureus*.

In recent years, USA400/J has been the predominant MRSA strain isolated from Japanese hospitals; however, few reports have analysed its phylogenetic characteristics [41]. Therefore, we performed a phylogenetic analysis based on whole-genome SNPs to compare the USA400/J isolates in this study with the ST1-IV strains isolated overseas (Figure 2). In the phylogenetic tree, the PVL-positive ST1-IV strains from the USA, Canada and Brazil formed a common cluster. These were closely related to USA400, MW2 and USA400-0051. Among the PVL-negative ST1-IV strains, those from Europe (such as Germany and France) belonged to a common cluster. In contrast, all USA400/J isolates from Japan formed a distinct cluster. These results showed that the CC1-IV strains isolated in Japan form a unique cluster, separate from ST1-IV strains found elsewhere. The number of SNPs, which indicate genetic differences, was compared between the strains. AM21, AM23 and AM25 differed by only one or two SNPs, suggesting they are closely related clones. In contrast, AM15 showed 104–105 SNP differences from these strains, indicating it is genetically distinct and unlikely to be part of the same outbreak. The outbreak strains isolated in this study (AM21, AM23 and AM25) were similar to those isolated in Tokyo, Japan (59621, 59731, 59736, 59553, 59458, 59574, 59482, 59691, TUM17881, TUM17988, TUM17904 and TUM17909) and strains isolated in Shiga and Kyoto (SUM816 and SUM924), with 89–113 SNPs. However, they shared 77–88 SNPs with the outbreak strains isolated in the NICU in Chiba, Japan (CN02, CN03, CN05, CN06, CN07, CN08, CN09, CN11, CN13, CN14, CN17 and CN25) and showed the most similar characteristics among previously reported genomic data.

## Discussion

This study analysed the MRSA outbreak strains that occurred in the NICU of a university hospital. The results showed that the outbreak was caused by MRSA strains of ST1-IV and ST764-II genotypes. Phylogenetic analysis using WGS of ST1-IV (USA400/J) revealed phylogenetically distinct features compared with CC1-IV strains isolated overseas.

ST1-IV and ST764-II are the representative MRSA genotypes reported in Japanese hospitals [15,41,42]. Both strains isolated in this study lacked virulence factors such as PVL and ACME, which is consistent with the characteristics from previous reports [13,43]. The ST764-II strain was first reported in 2007 as a mutant of the ST5-II strain and has since been isolated from various medical institutions in Japan [41,44]. In recent years, epidemics have also been confirmed in other regions of Asia, including China and Thailand [45,46]. In China, the detection rate of ST764-II strains has increased annually, and recent reports describe an epidemic of a highly virulent clone with characteristics similar to those of the Japanese ST764-II strain [46,47]. The relationship between these strains and the ST764-II isolate in this study is not yet known, and further detailed analyses based on genomic data are required. The isolation dates of AM20 (ST764-II) and AM15 (ST1-IV) differed by approximately three months from those of other strains with the same genotype. Therefore, although AM15 showed high homology using PFGE, it was considered to have low relevance to this outbreak. Genomic analysis of USA400/J revealed that AM15 differed significantly from other ST1-IV strains with respect to the number of SNPs. As a result, AM15 is thought to have caused NICU infections independent of the current outbreak. MRSA can spread in hospitals via cross-infection [48,49]. Therefore, transmission in NICUs is thought to occur from one affected infant to another via healthcare workers acting as a source of infection. In this study, MRSA contamination was found in areas where hands and fingers commonly come into contact, such as incubators and computers. This suggests that when a child is infected with MRSA, the surrounding area becomes contaminated, particularly in places frequently in contact with hands. MRSA is then spread through repeated contact infections involving peripheral equipment and medical staff. A report by Yamazaki *et al.* in 2024 suggested that some MRSA strains adapt to the hospital environment by regulating the accessory gene regulator (Agr) through genomic cytosine methylation [19]. The USA400/J isolate in this study was also phylogenetically similar to these related strains. However, whether it underwent epigenetic changes under specific environmental conditions remains to be determined.

The USA400/J isolated in this study showed high homology to USA400 using PFGE. However, USA400/J did not possess PVL, which is a toxic characteristic of USA400. Therefore, USA400/J was considered a different strain from USA400. Several Japanese studies reported CC1-IV strains with the same characteristics as USA400/J [13,17,18]. Therefore, it is possible that USA400/J is endemic to different regions of Japan. CC1-IV strains are reportedly endemic to Europe and Australia [50,51]. WA-1, which is endemic to Western Australia, and USA400/J cannot be distinguished from USA400 using standard typing methods [51]. Therefore, we performed a phylogenetic analysis of USA400/J and CC1-IV strains isolated overseas using previously available WGS data. The results showed that CC1-IV strains from Europe, Australia and Japan formed their own clusters. Phylogenetic analysis suggested that the CC1-IV strains from Japan and Australia were prevalent and evolved independently in both countries. It was also shown that USA400/J has phylogenetically distinct characteristics from those of previously reported USA400. Notably, USA400/J and USA400 differed in the presence or absence of PVL, which is difficult to detect using conventional PFGE. Therefore, a detailed analysis using WGS is required to clarify the prevalence of USA400/J in hospitals.

The main clinical significance of our study is that the USA400/J clone can become established in hospital environments such as NICUs and is highly prone to nosocomial transmission. Additionally, Japanese USA400/J isolates constitute a distinct phylogenetic cluster that is PVL negative and diverges from conventional USA400. This suggests that treatment strategies and infection control measures based on overseas MRSA data may not be directly applicable in Japan. These findings highlight the importance of tailoring therapeutic and infection control strategies to the characteristics of MRSA clones circulating domestically.

This study had several limitations. The study was unable to investigate the bacterial carriage status of the affected children and medical staff in the NICU. It also did not include an analysis of MRSA isolates from the environment. In addition, it was not possible to accurately determine the type, frequency or performance of the procedures conducted in affected children from whom MRSA was isolated. Consequently, the source and route of MRSA transmission in the NICU could not be identified. Several outbreaks involving strains with USA400/J characteristics have been reported in Japan. However, the available WGS data were limited and could not be included in this study. Therefore, larger comparative genomic analyses are required to elucidate the origin and evolution of USA400/J.

In this study, conventional methods and WGS revealed an outbreak of USA400/J infection in a NICU. This study suggests that USA400/J is a phylogenetically distinct clone from the ST1-IV strains isolated overseas and is spreading specifically in Japanese hospitals. Outbreaks of USA400/J infections have been identified in several Japanese hospitals. Therefore, the findings highlight the need for increased surveillance and infection control measures targeting USA400/J.

## CRediT authorship contribution statement

**Takuma Yoshida:** Writing – original draft, Visualization, Software, Methodology, Investigation, Formal analysis, Data curation. **Yuka Yamagishi:** Writing – review & editing, Resources, Data curation, Conceptualization. **Hiroshi Kaneko:** Visualization, Validation, Software, Methodology, Investigation, Formal analysis, Data curation. **Shunsuke Takadama:** Methodology, Investigation, Formal analysis, Data curation. **Hiroshige Mikamo:** Writing – review & editing, Supervision, Resources. **Hidemasa Nakaminami:** Writing – review & editing, Validation, Supervision, Project administration, Funding acquisition, Conceptualization.

## Data availability statement

All sequence data generated in this study have been deposited in the Sequence Read Archive (DNA Data Bank of Japan BioProject ID: PRJDB20266).

## Funding sources

No funding was received for this study.

## Conflict of interest statement

None declared.
